# A novel TaqMan probe-based qPCR method for rapid detection of the bacteria-associated amoeba *Heterostelium pallidum*

**DOI:** 10.1128/spectrum.01311-25

**Published:** 2026-04-20

**Authors:** Da Sun, Jianhua Ge, Jialing Jin, Xiangrui Xie, Yanxi Long, Ying Li, Zhuang Li, Steven L. Stephenson, Yu Li, Pu Liu

**Affiliations:** 1 Engineering Research Center of Edible and Medicinal Fungi, Ministry of Education, Jilin Agricultural University 85112, Changchun, China; 2 Shandong Provincial Key Laboratory for Biology of Vegetable Diseases and Insect Pests, College of Plant Protection, Shandong Agricultural University 34734, Tai’an, China; 3 Department of Biological Sciences, University of Arkansas 3341, Fayetteville, Arkansas, USA; Luonnonvarakeskus, Oulu, Finland

**Keywords:** dictyostelid cellular slime molds, environmental microorganism, microbiological rapid detection, TaqMan probe, qPCR

## Abstract

Dictyostelid cellular slime molds (dictyostelids) exhibit complex interactions with bacteria and have become an important research tool for studies of the relationships that exist between the ecological environment and pathogenic microorganisms. *Heterostelium pallidum*, a species of dictyostelid, is the type species of the genus *Heterostelium*. Traditional methods for isolating *H. pallidum* are time-consuming and ineffective. In the present study, a pair of specific primers and probes based on the genome of *H. pallidum* was designed and screened, optimized reaction conditions were determined, and a real-time fluorescence quantitative PCR detection method was developed. A standard curve was constructed with concentrations ranging from 1 ng/μL to 10 fg/μL. These yielded a correlation coefficient of 0.996 and an amplification efficiency of 103.354%. This method was tested and found to work for three random environmental soil samples. An effective CT value was detected in a soil sample where only a single clone of *H. pallidum* was isolated. The TaqMan probe-based fluorescence quantitative PCR method demonstrated high sensitivity and accuracy. The results obtained in this study have improved the limitations of traditional isolation methods used for *H. pallidum*, and these can be used for the rapid detection and determination of *H. pallidum* in different environments, providing reference data for research on the soil environment, microbial assemblages, and public health.

**IMPORTANCE:**

*Heterostelium pallidum* has been widely used in studies of chemotaxis and signaling in organisms as well as in other aspects of cell biology. However, its ecological relationships within diverse soil bacterial communities and its broader environmental roles remain poorly characterized, unlike the extensively studied model organism *Dictyostelium discoideum*. The question of how to enhance the value of *H. pallidum* in ecology through the application of specific technological approaches is a seriously understudied topic. Based on this situation, the present study developed a quantitative detection method for *H. pallidum*. Efficient detection of this organism in soil samples validated the applicability of this method for complex environmental samples. The specific probe developed in this study for *H. pallidum* changes the situation of time-consuming and low sensitivity associated with traditional methods of isolation, thus providing technical references for the application of *H. pallidum* in a wide range of ecological and environmental research.

## INTRODUCTION

Environmental microorganisms constitute an integral component of both natural and human habitats and fluctuations in their communities have profound impacts on ecological systems and public health (1, 2). The composition of microbial communities is constrained by spatiotemporal factors, resulting in distinct assemblages being associated with different environmental situations (3, 4). Soil ecosystems harbor diverse protist communities that maintain intimate associations with bacteria. These protists play pivotal roles in soil ecological cycles by mediating coordination among various species of microbes and influencing plant growth and health through complex biotic interactions (5).

Dictyostelids are a group of organisms belonging to the kingdom Protista, phylum Amoebozoa, and class Dictyostelia. They occupy a wide range of habitats but mainly occur in the litter layer of soil, although they can also grow in water or at the solid-liquid interface formed by water and soil (6, 7). For example, the model organism *Dictyostelium discoideum*, a species of dictyostelid, has also been found in urban wastewater and tap water (8–10). Dictyostelids obtain nutrients primarily by preying on bacteria, and most bacteria are subject to predation. Therefore, dictyostelids are one of the natural enemies of bacteria (11–13). However, the relationships between dictyostelids and bacteria are complex. In addition to the predation relationship, some bacteria display a staged symbiosis with dictyostelids. Examples such as *Legionella pneumophila*, *Pseudomonas aeruginosa*, and *Paraburkholderia* spp. have the ability to resist the predation of dictyostelids and even use this group as a refuge to avoid adverse environments (14–21). Brock et al. (22) observed that approximately one-third of the sorocarps of *D. discoideum* they isolated produced bacteria through sporulation and diffusion processes. The phenomenon of agricultural behavior in *D. discoideum* was discovered, demonstrating the close relationship between dictyostelids and bacteria (23, 24). Therefore, dictyostelids can serve as a potential pathway for bacterial and even pathogenic transmission. Collectively, these findings suggest that dictyostelids exhibit significant potential for applications in environmental monitoring and biological control. Historically, *Heterostelium pallidum* was investigated alongside other related species of dictyostelids in comparative studies. However, current research targeting this organism in ecological studies is limited due to persistent taxonomic ambiguities and technical challenges in maintaining axenic cultures. Therefore, establishing a detection method for *H. pallidum* in soils holds scientific significance for the expansion of the research area on this protist.

Dictyostelids usually exist as single-cell forms. When subjected to starvation, they aggregate into multicellular mucinous structures, gradually forming sorocarps and producing spores. The latter is also one of their main biological features (25). At present, this feature is commonly used to collect separable individuals from environmental samples, and the 18S rDNA, which encodes the small subunit (SSU) rRNA gene region, is amplified by conventional PCR, followed by sequencing to obtain the sequence data of the sample, which are combined with morphological alignment for final identification (26–28). At an early stage, it is necessary to first prepare a suspension of the collected soil for separation to obtain the separable individuals (29). Although dictyostelids are relatively common in natural environments, their individual vegetative cells are small and scattered. The steps used for their isolation often need to be repeated multiple times to obtain the strains present. Subsequently, the strains obtained are purified and subjected to repeated large-scale collection of sorocarps to meet the requirements for DNA extraction. The entire separation process is complicated and requires considerable time to carry out. Although the use of this method to amplify DNA fragments has universal applicability within the dictyostelids, it cannot quickly and accurately be used to obtain a single strain and does not meet the needs of rapid sample screening.

**Fig 1:**
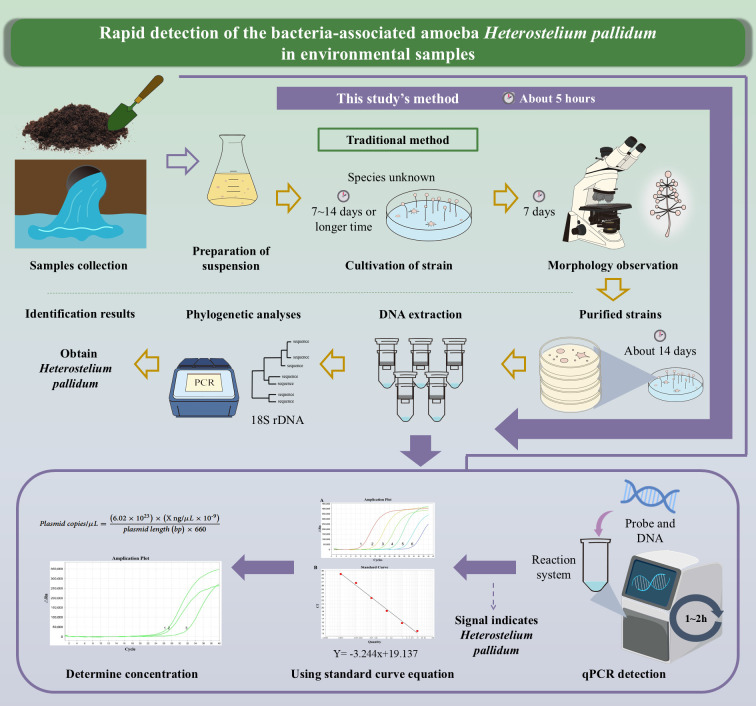
Comparison of the study described in this paper and the traditional method used for isolating *Heterostelium pallidum* from environmental samples.

Quantitative real-time PCR (qPCR) is a well-established technique for rapid and accurate quantification of nucleic acids. The probe method only needs one to design a specific probe in the PCR system to detect the fluorescence signal in real time under fully closed conditions (30). The TaqMan probe is the most widely used hydrolysis probe (31). Fluorescence quantitative PCR detection using the probe method is accurate, rapid, and simple to operate. This method has been widely applied to the rapid detection of biological samples, including fish, pathogens, and viruses (32–34). It cannot only assist clinical medicine in rapid detection but can play an important role in food safety detection and even has many applications in environmental health monitoring, customs, and biological detection (30, 31, 35, 36). Therefore, by designing the specific primers of *H. pallidum* and using the probe method for real-time fluorescence quantitative PCR to establish a standard curve, it can overcome the shortcomings of existing technologies and establish a rapid, highly specific, and easy-to-operate quantitative detection of *H. pallidum*. The method realizes the rapid screening of *H. pallidum* and effectively shortens the repeated steps of screening the target dictyostelids in different environments (Fig. 1).

## RESULTS

### Screening specific primers for *H. pallidum*

**Fig 2:**
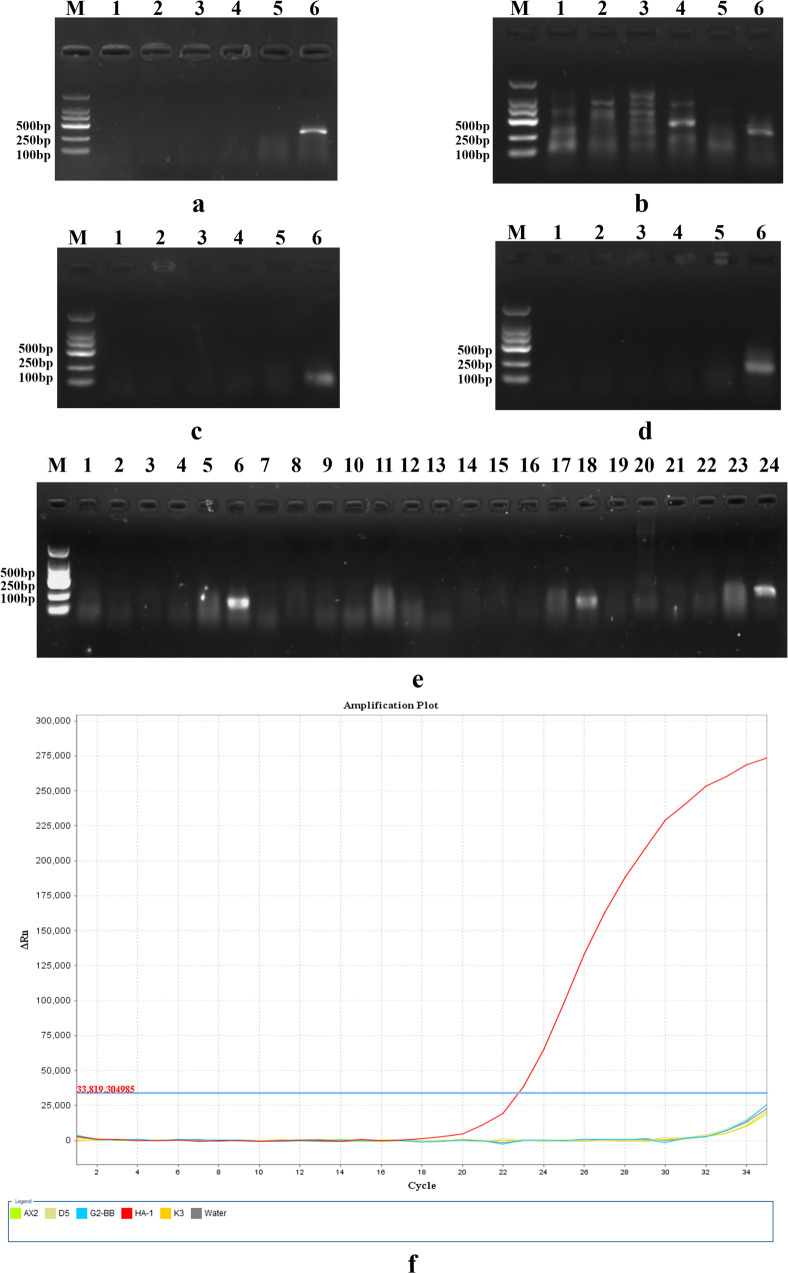
Results of conventional PCR amplification with primer specificity. (**a**) HA7F/R; (**b**) HA8F/R; (**c**) HA2F/R; (**d**) HA3F/R; and (**e**) 1–6: HA12F/R; 7–12: HA1F/R; 13–18: HA5F/R; and 19–24: HA6F/R. (**a–e**) M: DNA marker 2,000 bp, (**a–d**) lanes 1–6: Water, D5, K3, G2-BB, AX2, and HA-1. (**e**) Water: 1, 7, 13, 19; D5: 2, 8, 14, 20; K3: 3, 9, 15, 21; G2-BB: 4, 10, 16, 22; AX2: 5, 11, 17, 23; and HA-1: 6, 12, 18, 24. (**f**) qPCR specificity validation of the TaqMan probe and primers for *Heterostelium pallidum*. Amplification curves are shown for: *H. pallidum* (HA-1), other dictyostelid species (D5, K3, G2-BB, and AX2), and negative control (water). The *x*-axis ends at Cycle 35.

The designed 12 pairs of primers were subjected to gradient amplification using the DNA of *H. pallidum* as a template. The results showed that primers HA1F/R, HA2F/R, HA3F/R, HA4F/R, HA5F/R, HA6F/R, HA7F/R, HA8F/R, and HA12F/R were the qualified primers for the initial screening (Fig. S1). Further specific screening was performed on the primers that passed the initial screening. Specific primer pairs were subjected to routine PCR with the genomes of water (negative control), D5, K3, G2-BB, AX2, and HA-1 (Fig. 2). Electrophoretic analysis revealed distinct primer performance: the HA8F/R primer pair amplified target sequences across all tested dictyostelid species (Fig. 2b), whereas HA1F/R failed to produce detectable amplification products in any dictyostelid strains under identical PCR conditions (Fig. 2e). The target band of gel electrophoresis of HA2F/R and HA3F/R was lighter. Based on the amplification band status, HA7F/R (Fig. 2a) was selected as the specific primer pair for this experiment.

We further evaluated the specificity of the designed primers and probe using qPCR (Fig. 2f). The results demonstrated that the TaqMan probe and primer set produced a distinct amplification signal exclusively for *H. pallidum* (HA-1). In contrast, no significant fluorescence signal (CT value > 35) was detected in reactions containing DNA from other dictyostelid species (D5, K3, G2-BB, and AX2) or the negative control (water), confirming the high specificity of the assay for *H. pallidum*.

### qPCR reaction conditions and standard curve

**Fig 3:**
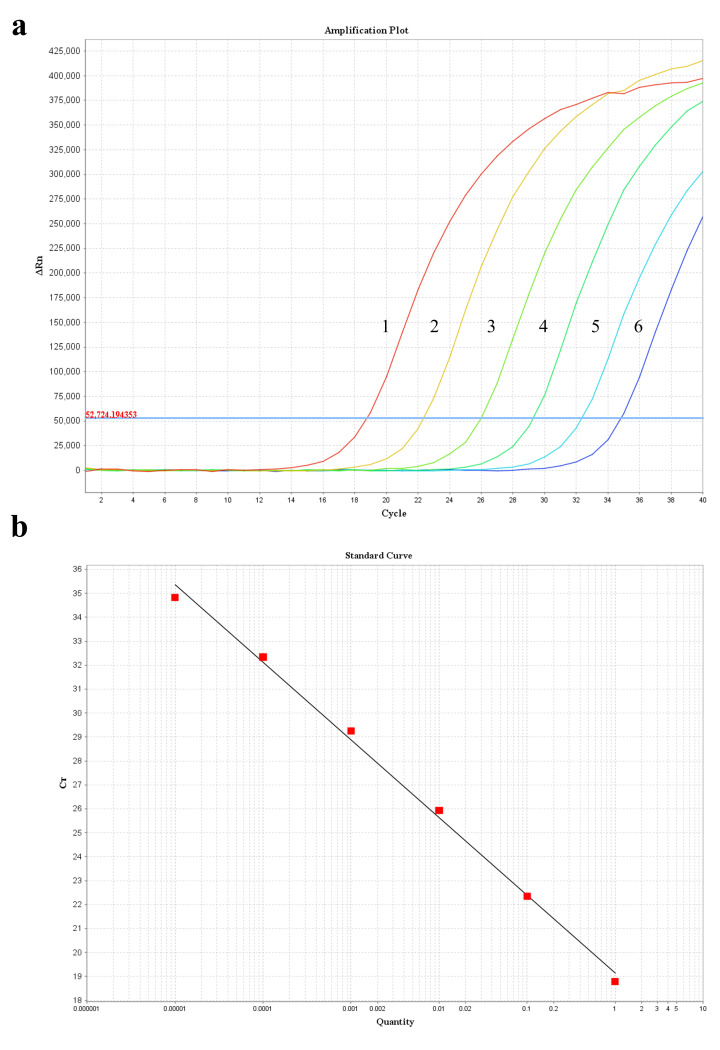
Amplification curve and standard curve of plasmid standards. (**a**) Standard amplification curve, 1–6: 1 ng/μL, 100 pg/μL, 10 pg/μL, 1 pg/μL, 100 fg/μL, and 10 fg/μL. (**b**) *H. pallidum* plasmid standard curve.

The fluorescence quantitative reaction can achieve good amplification in the range of 1 ng/μL to 10 fg/μL under a probe concentration of 100 nmol/L, a primer concentration of 400 nmol/L, an annealing temperature of 55°C, and a total reaction system volume of 20 μL (Fig. 3a).

According to the linear relationship between the log (*x*) of DNA concentration and the corresponding CT value (*y*), an equation was obtained:


(1) Y=−3.244x+19.137

*R*^2^＝0.996 and the amplification efficiency was 103.354% (Fig. 3b).

### Determination of the limit of detection

**Fig 4:**
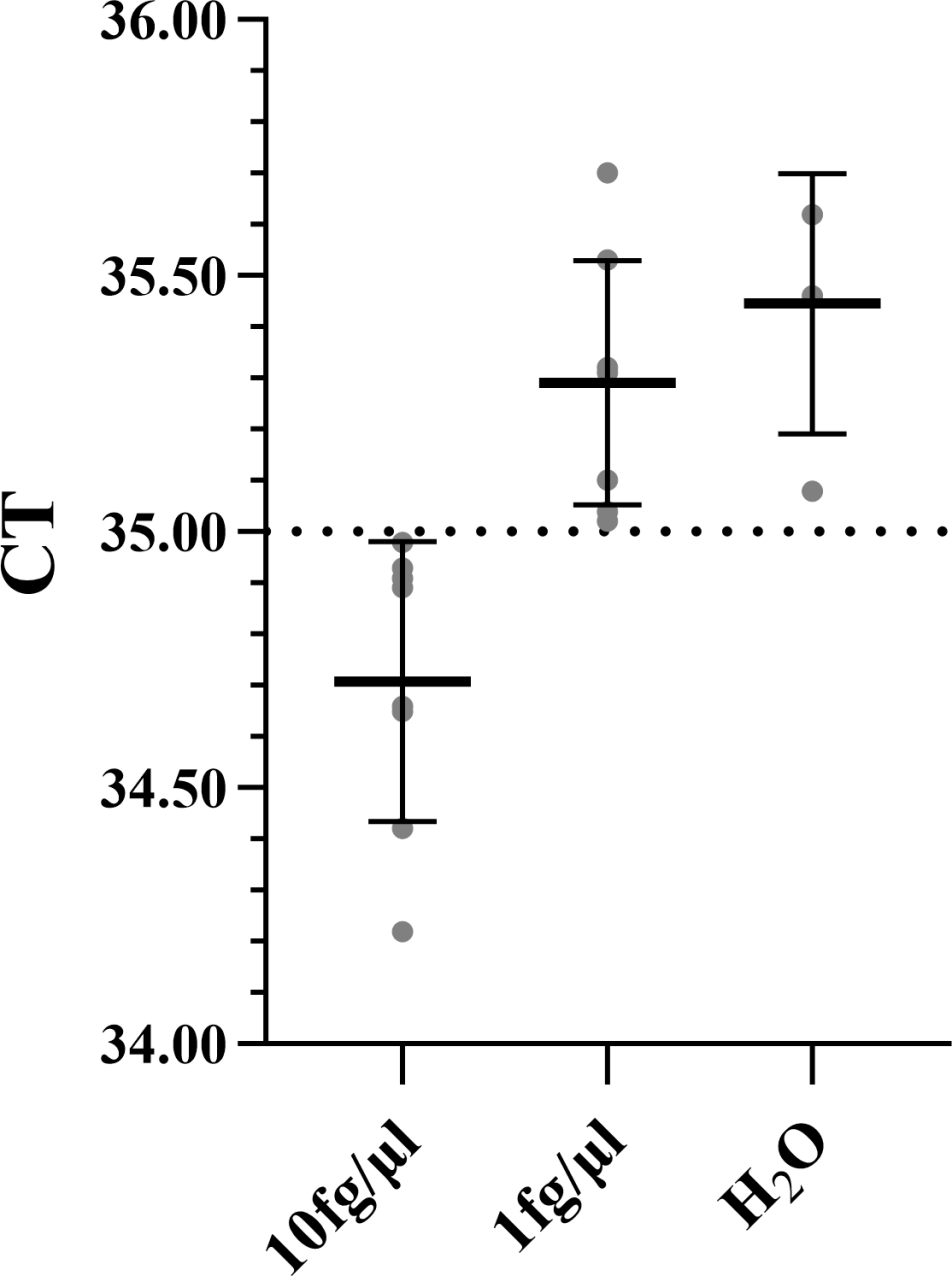
CT values for sensitivity evaluation. The plot shows the mean CT values (±SD) obtained from qPCR runs of plasmid standards at concentrations of 10 and 1 fg/μL, along with the negative control (ddH_2_O). The CT values for the 1 fg/μL group consistently exceeded 35 and were statistically indistinguishable from those of the negative control. These results confirm that 10 fg/μL is the lower limit of quantification for this assay, with samples yielding CT > 35 defined as “not detected.”

To further evaluate the sensitivity of the developed method, replicate qPCR assays were performed on the two lowest concentrations (10 and 1 fg/μL) of the plasmid standard curve as well as on a negative control (ddH_2_O). The results (Fig. 4) indicated that all replicates of the 1 fg/μL concentration group yielded CT values exceeding 35, with amplification curves indistinguishable from those of the negative control (water). This demonstrates that 1 fg/μL falls below the reliable detection limit of the assay. Consequently, 10 fg/μL was established as the lower limit of quantification for this method. Samples with CT values > 35 were considered “not detected.” This criterion was applied consistently for the detection of all subsequent environmental samples.

### Detection of different DNA concentrations of *H. pallidum*

**Fig 5:**
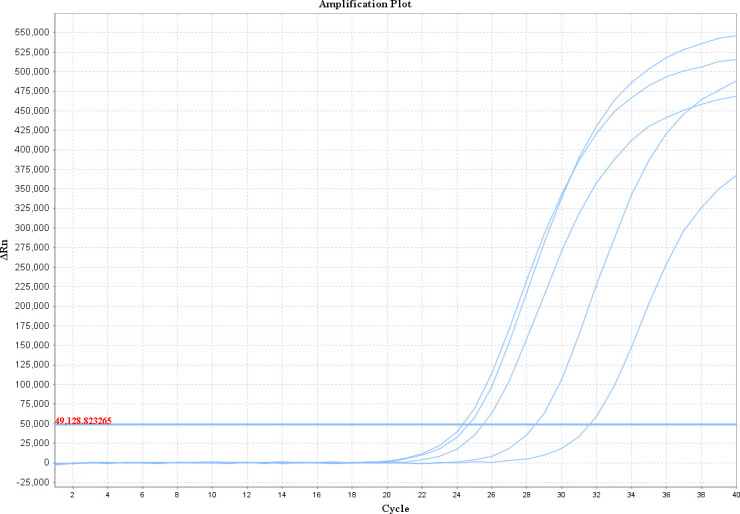
Amplification curves of qPCR for different concentrations of *H. pallidum*.

qPCR was performed on samples with known cell concentrations of *H. pallidum*, yielding characteristic S-shaped amplification curves (Fig. 5). The corresponding CT values and calculated copy numbers are summarized in Table 1. Using the standard curve (Equation 1) and plasmid copy number conversion (Equation 2), the average copy number corresponding to 1 × 10^6^ cells/mL was determined to be 3.41 × 10^5^ copies/μL. The detected cell concentrations were then derived by dividing the copy number obtained from CT values by the average copy number (Table 1).

**TABLE 1:** Cell concentration and copy number of *H. pallidum*

Cell concentration (×10^6^ cells /mL)	Average CT value	Copies/μL	1 × 10^6^ cells/mL correspond to copies/μL
20	24.33	5.34 × 10^6^	2.67 × 10^5^
10	24.7	4.11 × 10^6^	4.11 × 10^5^
5	25.52	2.30 × 10^6^	4.59 × 10^5^
1	28.53	2.71 × 10^5^	2.71 × 10^5^
0.1	31.64	2.98 × 10^4^	2.98 × 10^5^

### Suspension sample detection of *H. pallidum*

**Fig 6:**
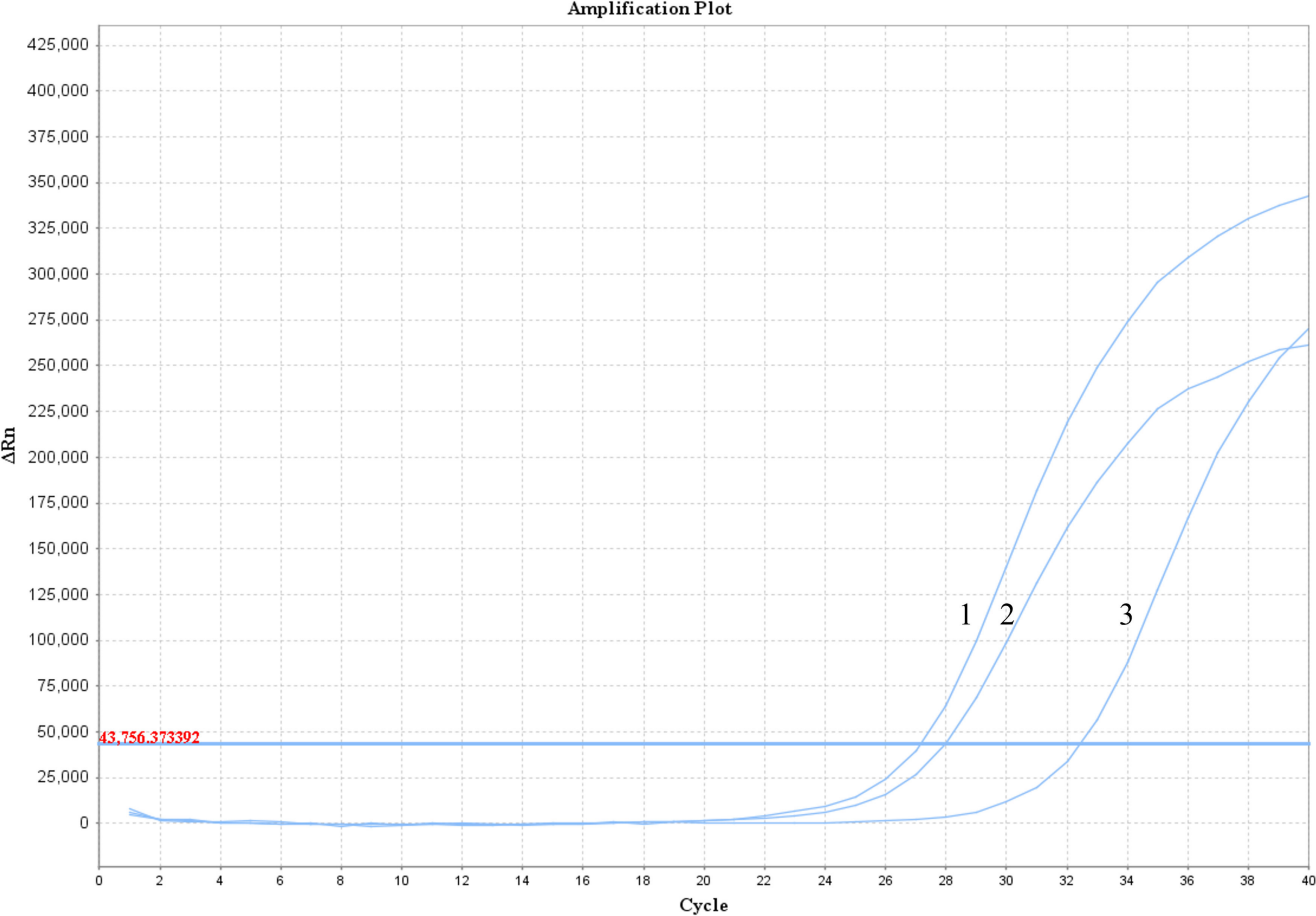
Suspension samples amplification curve of *H. pallidum*.

Three suspension samples of *H. pallidum* were selected and the cell concentration was determined; cell concentrations were 5 × 10^6^, 2 × 10^6^, and 1 × 10^6^ cells/mL. DNA from these samples was extracted and subjected to qPCR detection. The amplification results showed that these samples could be amplified (Fig. 6).

CT values were matched with copy numbers to further obtain the cell concentration in the random samples of *H. pallidum* (Table 2).

**TABLE 2:** Suspension samples cell concentration and copies of *H. pallidum*

Number	CT value	Copies/μL	Detected cell concentration (×10^6^ cells/mL)	Determined cell concentration (×10^6^ cells/mL)
1	27.15	7.22 × 10^5^	2.12	5
2	27.95	4.09 × 10^5^	1.20	2
3	32.47	1.65 × 10^4^	0.049	1

A detection method using fluorescence quantitative PCR can quickly detect suspension samples and obtain the corresponding cell concentration.

### Isolation and identification of soil dictyostelids

**Fig 7:**
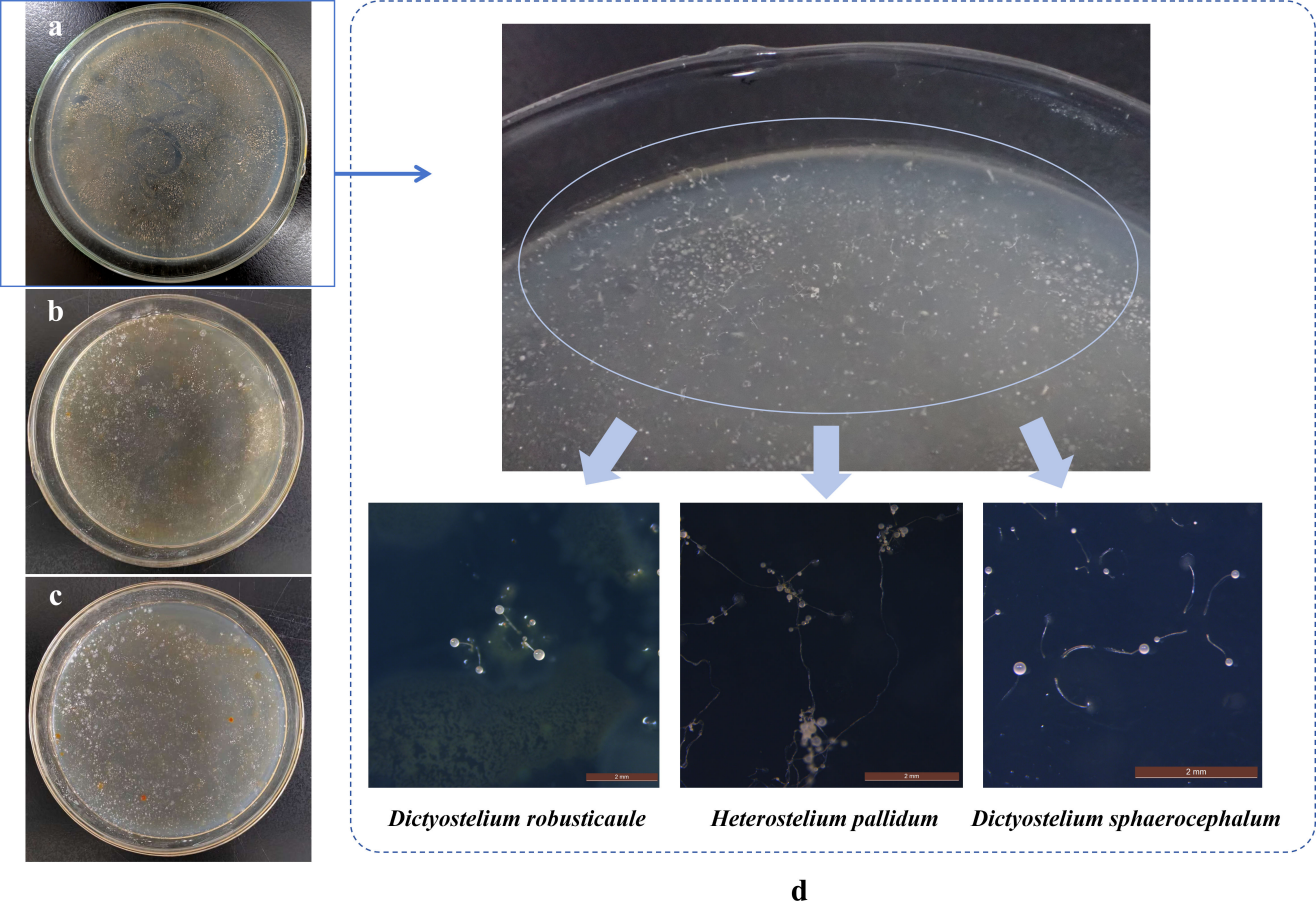
The soil suspension was cultured for dictyostelid isolates on the culture medium. (**a**) The number 7753 soil; (**b**) the number 7082 soil; (**c**) the number 7858 soil; and (**d**) soil sample (No. 7753) with qPCR positive and the dictyostelid isolates. Scale bars = 2 mm.

**Fig 8:**
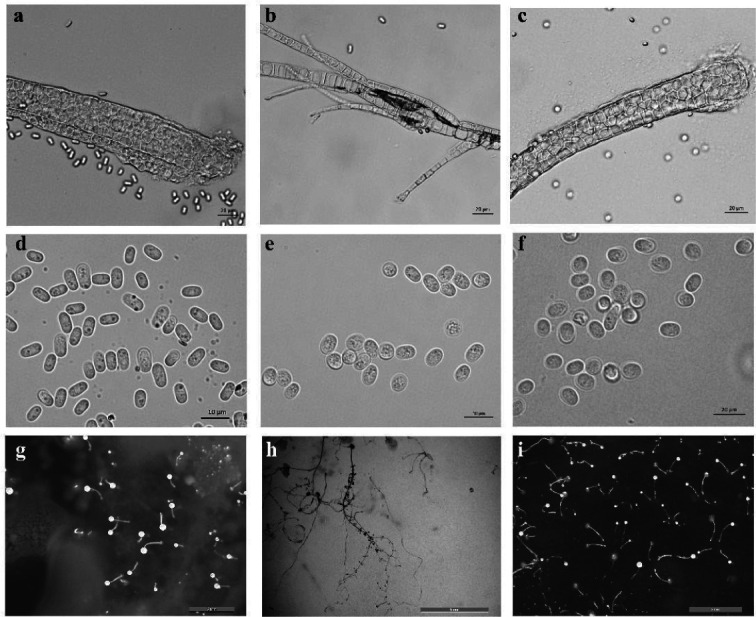
Morphological characteristics of three dictyostelids isolated in the present study. *D. robusticaule*: (**a**) sorophore base, (**d**) spores, (**g**) sorocarps; *H. pallidum*: (**b**) sorophore and sorophore tip, (**e**) spores, (**h**) sorocarps; *D. sphaerocephalum*: (**c**) sorophore base, (**f**) spores, (**i**) sorocarps. Scale bars: (a–c) = 20 µm; (d and e) = 10 µm; (f) = 20 µm; and (g–i) = 2 mm.

The conventional isolation procedures were carried out on the three soil samples, and cultivable dictyostelids observed only in the number 7753 soil (Fig. 7a). In the isolated medium of this soil sample, only one dictyostelid clone was obtained. The samples in this clone were purified, and three species of dictyostelids were identified; one of these was *H. pallidum* (Fig. 7d and 8). The descriptions of the three species are given below.

**Fig 9:**
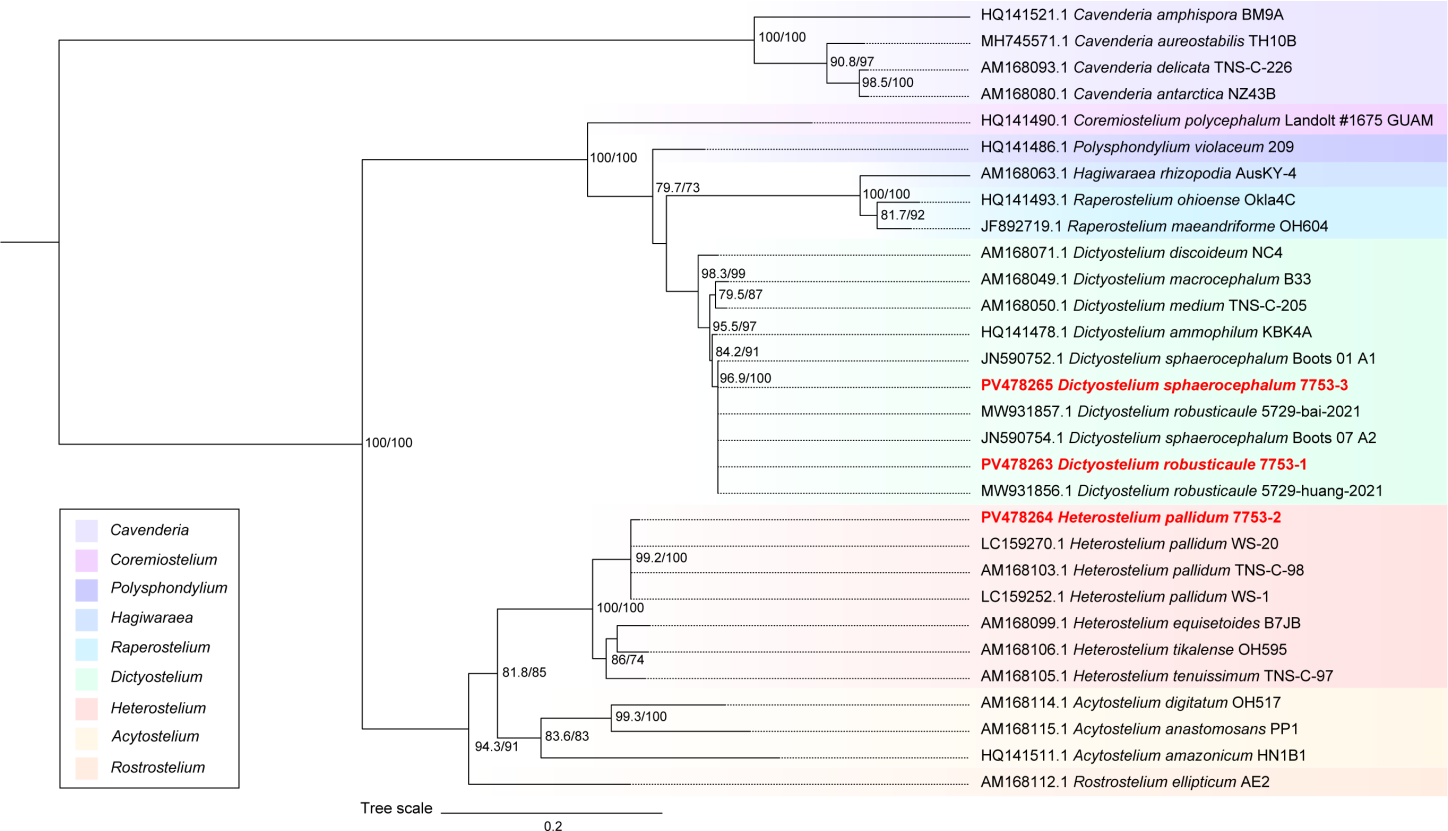
Phylogenetic tree of three isolated samples. Red marks are dictyostelids in the number 7753 soil sample. 7753-1 is *D. robusticaule*, 7753-2 is *H. pallidum*, and 7753-3 is *D. sphaerocephalum*.

We performed PCR using the primers 18S-FA and 18S-FB and obtained an approximately 1,800 bp rDNA fragment. Meanwhile, the alignment of three dictyostelids was performed in the phylogenetic tree (Fig. 9). Although *D. robusticaule* (7753-1) and *D. sphaerocephalum* (7753-3) clustered within a single branch on the phylogenetic tree. The taxonomic results (Table 3) based on morphology and phylogeny of the isolated samples were *D. robusticaule*, *H. pallidum*, and *D. sphaerocephalum*.

**TABLE 3:** Molecular and morphological identification of dictyostelids recovered from soil sample

Sample no.	Species name	GenBank accession	Morphology description
7753-1	*Dictyostelium robusticaule*	PV478263	Spores oblong, with polar granules; Sorocarps stout and polymorphic, sorophore apex with a prominent collar.
7753-2	*Heterostelium pallidum*	PV478264	Spores smooth, oval or nearly spherical, without polar granules; Sorocarps clustered, stout, sorophore with a collar.
7753-3	*Dictyostelium sphaerocephalum*	PV478265	Spores oblong, with unconsolidated polar granules; Sorocarps with regular whorls of branches, pseudoplasmodium migrating.

### Environmental soil sample detection of *H. pallidum*

**Fig 10:**
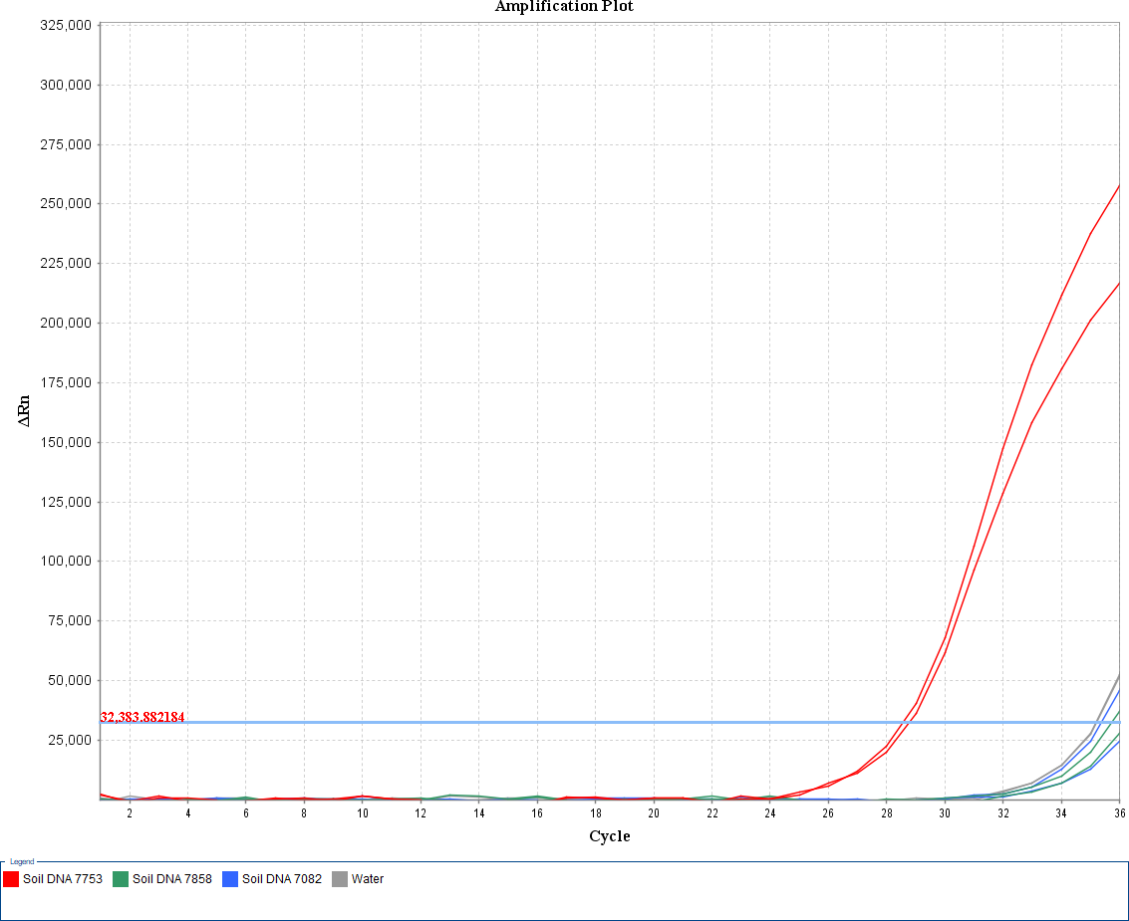
The DNA samples' amplification curve of *H. pallidum*.

All soil samples collected for extraction of DNA were analyzed through qPCR with TaqMan probes (Fig. 10). The water was used as a negative control. The qPCR results demonstrated that only the DNA extraction of soil No. 7753 had an effective CT value, with the negative control and the two other soil DNA (Nos. 7082 and 7858) reactions exhibiting CT values > 35, thus the results were negative (Table 4).

**TABLE 4:** Cell concentration and copy number of *H. pallidum* in DNA samples

Number	Average CT value	Copies/μL	×10^6^ cells/mL	Type	Clone region
1	28.70	2.41 × 10^5^	0.706	Soil DNA 7753	1
2	35.97	-^*b*^	-^*b*^	Soil DNA 7082	0^*a*^
3	35.89	-^*b*^	-^*b*^	Soil DNA 7858	0^*a*^
4	35.17	-^*b*^	-^*b*^	Water	0^*a*^

^*a*^ *Heterostelium pallidum* was not isolated from the clone regions of samples.

^*b*^ CT value > 35 was considered a negative result and could not be quantitatively analyzed.

To evaluate potential PCR inhibition in the extracted soil DNA, a spike-in recovery experiment was performed. A known concentration of *H. pallidum* plasmid standard was added to the DNA extracts from soil samples 7082 and 7858 (qPCR-negative samples), as well as to nuclease-free water as a control. The analysis (Table S3) showed no significant difference in CT values between the spiked soil DNA samples and the control, confirming the absence of significant PCR inhibition in these samples. Therefore, the negative qPCR results for soils 7082 and 7858 were due to the absence of target DNA rather than inhibition.

Among the three randomly selected soil samples, only one sample displayed CT values within the suitable quantification range, which was consistent with the result from conventional isolation method. Through standard curve calibration, the viable *H. pallidum* cell concentration in this positive sample was calculated as 7.06 × 10^5^ cells/mL.

## DISCUSSION

In this paper, we describe the development of a fluorescence quantitative qPCR detection method that can be used to achieve rapid detection of *H. pallidum*. The lowest detection concentration on the standard curve was 10 fg/μL. As a preliminary validation of its applicability to complex environmental matrices, this method was tested on three randomly selected environmental soil samples. In one of these samples, an *H. pallidum* clone was isolated and successfully detected, confirming the method’s functionality.

Our newly developed qPCR method effectively addresses these limitations. Conventional approaches that depend on morphological observation and isolation encounter challenges in rapidly identifying and screening of dictyostelids within large-scale environmental samples. Compared to conventional 18S rDNA amplification methods, our protocol enhances the accuracy and shortens the time of species identification. Different vegetation types, environmental temperatures, soil physicochemical properties, decomposable organic matter, and bacterial communities significantly influence the composition of the dictyostelid community in specific soil environments (6, 28, 37). The quantity and diversity of dictyostelids also reflect the diversity of other organisms in the environment (29, 38, 39). In this study, our qPCR method successfully detected *H. pallidum* in an environmental soil sample, from which a corresponding clone was isolated. This validates the method’s practical utility for environmental monitoring and demonstrates its potential to accelerate research into the ecological roles of dictyostelids.

Although TaqMan probe-based qPCR is prevalent in detecting bacteria, fungi, and viruses (40–44), its application to dictyostelid research has been unreported. Our study fills this gap by establishing the first such assay for *H. pallidum*. The high sensitivity and specificity demonstrated here provide a powerful tool not only for direct detection and quantification but also for probing its ecological roles. Given that dictyostelids can act as bacterial vectors and environmental refugia for pathogens (16, 19, 45–47), the ability to rapidly monitor their presence offers a promising indirect approach for assessing microbial dynamics and biosafety risks. The positive results from our initial environmental tests warrant further investigation across a wider range of sample types and conditions.

It is important to note that the cell concentrations determined in this study are semi-quantitative estimates derived from a plasmid DNA standard curve. While this approach provides a robust and reproducible means for rapid screening and comparative analysis of *H. pallidum* presence across samples, absolute quantification in complex environmental matrices would require additional calibration to account for variables such as DNA extraction efficiency and potential PCR inhibition.

In summary, this study presents a novel qPCR assay for *H. pallidum* and provides preliminary evidence of its effectiveness in environmental samples. This method enables rapid screening of the target organism and serves as a valuable reference technique for future studies on soil ecology, microbial community interactions, public health, and the complex relationships between dictyostelids and bacteria.

## MATERIALS AND METHODS

### Collection and culture of samples for dictyostelid species

All dictyostelid strains were preserved in the Herbarium of the Mycological Institute of Jilin Agricultural University (HMJAU), Changchun, China (Table 5). From them, *D. discoideum* and *H. pallidum* were suspension cultures, and the other strains were plate cultures. Species identification was performed using both conventional PCR amplification with taxon-specific primers and comparative morphological analysis (39, 48).

**TABLE 5:** Dictyostelid strains used for specificity testing and qPCR

Strain number	Species
AX2	*Dictyostelium discoideum*
D5	*Cavenderia delicata*
G2-BB	*Dictyostelium giganteum*
HA-1	*Heterostelium pallidum*
K3	*Polysphondylium* sp.

### DNA extraction of test strains

Genomic DNA was extracted from 2 mL aliquots of dictyostelid suspensions using the MiniBEST Universal Genomic DNA Extraction Kit (Takara Bio, Japan) according to the manufacturer’s protocol. The genomic DNA concentration was detected using a QUAWELL Q5000 spectrophotometer (QUAWELL, American) and the DNA solutions were stored at −20°C in double-distilled water for subsequent applications.

### Primers and PCR assay

Using Primer 5 and National Center for Biotechnology Information (NCBI) BLAST program to design specific primers for the genome of *H. pallidum* (49, 50), a total of 12 pairs of primers were designed (Table S1), with amplified fragments ranging in length from 100 to 300 bp. Subsequently, using the genome of *H. pallidum* as a template, qualified primers were screened for gradient amplification through conventional PCR. The total amount of the reaction system is 25 μL, 1 μL of it contained forward primer, 1 μL of reverse primer, 1 μL of DNA plate, and 12.5 μL of 2 × Magic Green Taq SuperMix, with ddH_2_O added to adjust the final volume to 25 μL. The reaction procedure was as follows: pre-denaturation: 95°C, 3 min; 35 cycles: denaturation: 95°C, 30 s; Annealing: 60–55°C, 30 s; Extension: 72°C, 1 min; and Final extension: 72°C, 5 min. The PCR amplicons were loaded onto 1% agarose gels containing 0.1% ethidium bromide and visualized under ultraviolet light. Using the test strain as a template and water as a negative control, perform specific PCR amplification and screen for specific primers. According to the amplification results, TaqMan qPCR primer probe was designed (Table S1).

The dictyostelids identification reaction was the conventional PCR system. The reaction procedure was as follows: pre-denaturation: 95°C, 5 min; 30 cycles: denaturation: 95°C, 30 s; Annealing: 56°C, 1 min; Extension: 72°C, 2 min; and Final extension: 72°C, 10 min. And the genomic DNA solution was used directly for PCR amplification using the forward primer 18S-FA (5′-AACCTGGTTGATCCTGCCAG-3′) and reverse primer 18S-FB (5′-TGATCCTTCTGCAGGTTCAC-3′) (51). PCR products were sent to Sangon Bio-tech Co., Ltd. (Shanghai, China), for sequencing.

### Preparation of plasmid standard for *H. pallidum*

The target fragment was amplified from the *H. pallidum* genome using specific primers, gel-purified, and ligated into the *pEASY*-T&B Zero Cloning Vector (TransGen Biotech, China). The ligation reaction was performed at 25°C for 10 min, followed by transformation into DH5α competent cells. Positive clones were verified by sequencing (Sangon Biotech, China). Plasmid DNA was extracted using a commercial kit, and its concentration was measured with a QUAWELL Q5000 spectrophotometer.

### Establishment of qPCR standard curve

The qPCR reactions were performed using TaqProbe 2× qPCR-Multiplex Mastermix (Sangon Biotech, China) in a 20 μL system containing 100 nmol/L probe, 400 nmol/L primers, and 1 μL DNA template. Cycling conditions were: 95°C for 30 s, followed by 40 cycles of 95°C for 15 s and 60°C for 30 s. The plasmid standard was serially diluted from 10 ng/μL to 1 fg/μL to generate the standard curve.

The limit of detection was determined as the lowest plasmid concentration that yielded a reproducible amplification curve with a CT value below 35 and could be consistently detected in all reactions.

### Data analysis and threshold setting

Raw fluorescence data were collected and analyzed using the StepOne Software v2.3 . The Ct for each reaction was automatically determined by the software. The threshold was set to intersect the amplification curves in the linear phase of exponential amplification, above the background fluorescence but below the plateau phase, in accordance with the software’s default algorithm. This algorithm positions the threshold above the background fluorescence signal but well below the plateau phase, ensuring an optimal and consistent signal-to-noise ratio for Ct determination across all runs within the same plate. The threshold line is visually indicated in all amplification curve figures.

### Detection for *H. pallidum* and conversion formula

To correlate cell concentration with DNA copy number, *H. pallidum* cell suspensions were prepared at concentrations of 2 × 10^7^, 1 × 10^7^, 5 × 10^6^, 1 × 10^6^, and 1 × 10^5^ cells/mL using a hemocytometer. Genomic DNA was extracted from 200 μL aliquots. qPCR was performed as described in “Establishment of qPCR standard curve” section. The plasmid copy number was calculated using the formula as follows (52):


(2) $$\text{ Plasmid copies }/\mu L=\left[\left(x\;ng/\mu L\times 10^{-9}\right)\times \left(6.02\times 10^{23}\right)\right]/[\text{ Plasmid length }(bp)\times 660]$$

The average copy number corresponding to 1 × 10^6^ cells/mL was derived, and cell concentrations in test samples were estimated by dividing the measured copy number by this average value.

### Suspension detection and quantification of *H. pallidum*

DNA was extracted from three liquid samples of *H. pallidum* cells, and the qPCR reaction system and conditions were used to obtain CT values. Utilizing the established standard curve, the CT value was correlated to the copy number. Given the known cell concentration of the standard, the average copy number was deduced through computation. The cell concentration was converted to the copy number by the detected CT value, and the final cell concentration obtained by dividing the copy number by the average copy number (53, 54).

### Soil sampling, isolation, and cultivation of dictyostelids

Samples used for isolation of dictyostelids were collected from the Inner Mongolia, Jilin Province, and Jiangsu Province, China in 2022 and 2023 (Table 6). All the soil samples were preserved in the HMJAU.

**TABLE 6:** Tested soil samples

Soil number	Soil type	Localities
7082	Grassland-chestnut soil	Inner Mongolia, China
7753	Farmland soil	Jilin Province, China
7858	Broad-leaved forest soil	Jiangsu Province, China

An improved version of Cavender and Raper (48) separation method was used. Sterile water was prepared by autoclaving distilled water at 121°C for 30 min. A soil suspension was created by homogenizing 10 g soil sample with 90 mL sterile water in a sealed flask using a constant temperature shaking table (23°C, 280 rpm/min) for 2 min, followed by 1 h sedimentation. Aliquots (500 μL) of the supernatant were aseptically spread onto hay agar plates. Incubated at 23°C for 7–14 days and monitored daily for microbial growth observation.

### Identification of dictyostelids isolation from soil

Both the morphology and phylogeny of the dictyostelid isolates recovered were subjected to detailed study (55). Dictyostelid isolates were identified with the use of the morphological descriptions provided by Index Fungorum, whose nomenclature also was followed except for those species recently assigned to new genera in the system of classification proposed by Sheikh et al. (27). The characteristic stages in the life cycle, including cell aggregation and the formation of pseudoplasmodium and sorocarps, were observed under a Zeiss dissecting microscope (Axio Zoom V16) with a 1.5× objective and 10× ocular. Slides with sorocarps were prepared with water as the mounting medium. Features of spores, sorophores, and sorocarps were observed and measured on the slides by using a Zeiss light microscope (Axio Imager A2), with 10× ocular and 10×, 40×, and 100× (oil) objectives. Photographs were taken with a Zeiss Axiocam 506 color microscope camera.

The DNA reaction information and the rDNA SSU sequence amplification used the primers 18S-FA and 18S-FB are described in “Primers and PCR assay” section. All the sequences were subsequently submitted to BLAST. All sequences of related species were downloaded from GenBank for phylogenetic analysis (Table S2) (56), and compared using ClustalW multiple alignment (57), and then subjected to a manual correction by MEGA 7.0 software (58). The calibrated sequences were performed with the IQTREE v.2.4.0 for maximum likelihood (ML) analysis, the evolutionary model is GTR+F I+G4 with 1,000 repetitions of bootstrap (59).

### Soil DNA extraction

The soil suspensions were subjected to vacuum filtration through 5 μm pore-size membrane filters. Membrane filters retained soil particulates, and the dictyostelid cells co-retained on the membrane surfaces were processed for genomic DNA extraction using the DNeasy PowerSoil Pro Kit (QIAGEN, Germany). The extracted DNA was preserved at −20°C for subsequent molecular analyses.

### Spike-in recovery experiment for PCR inhibition assessment

A known quantity of the *H. pallidum* plasmid standard was added to an aliquot of each environmental DNA extract that had tested negative in the initial qPCR screening (soils DNA 7082 and 7858). An equivalent amount of the same plasmid standard was also added to nuclease-free water as a non-inhibited control. The final concentration of the plasmid standard in all spiked samples was identical. These spiked samples were then subjected to qPCR using the assay conditions described in “Establishment of qPCR standard curve” section. The resulting CT values from the spiked soil DNA samples were compared to the CT value of the spiked water control.

## Data Availability

18S sequencing sequences from identified dictyostelids 7753-1, 7753-2, and 7753-3 are available in the GenBank under accession numbers PV478263, PV478264, and PV478265.

## SUPPLEMENTAL MATERIAL

Fig. S1 to S3; Tables S1 to S3 spectrum.01311-25-s0001.docx Fig. S1 to S3 contain the gel images and construction of standard plasmids. Tables S1 to S3 contain the primers, probes and sequences used in manuscripts.

ASM does not own the copyrights to Supplemental Material that may be linked to, or accessed through, an article. The authors have granted ASM a non-exclusive, world-wide license to publish the Supplemental Material files. Please contact the corresponding author directly for reuse.

## References

[1] Perfumo A, Çabuk U, Schulte L, Courtin J, Harms L, Stoof‐Leichsenring KR, Herzschuh U. 2023. Paleometagenomics reveals environmental microbiome response to vegetation changes in northern Siberia over the millennia. Environmental DNA 5:1252–1264. doi:10.1002/edn3.446

[2] Matthews K, Cavagnaro T, Weinstein P, Stanhope J. 2024. Health by design; optimising our urban environmental microbiomes for human health. Environ Res 257:119226. doi:10.1016/j.envres.2024.119226 38797467

[3] Bathia J, Miklós M, Gyulai I, Fraune S, Tökölyi J. 2024. Environmental microbial reservoir influences the bacterial communities associated with Hydra oligactis. Sci Rep 14:32167. doi:10.1038/s41598-024-82944-0 39741169 PMC11688501

[4] Sauer HM, Hamilton TL, Anderson RE, Umbanhowar CE, Heathcote AJ. 2022. Diversity and distribution of sediment bacteria across an ecological and trophic gradient. PLoS One 17:e0258079. doi:10.1371/journal.pone.0258079 35312685 PMC8936460

[5] Xu Q, Feng M, He B, Li T, Tang P, Zhang D, Xie Y. 2025. Soil micro-structure drives trophic interactions within micro-food webs via bottom-up regulation under different planting patterns. Agric Ecosyst Environ 383:109539. doi:10.1016/j.agee.2025.109539

[6] Zou Y, Liu P. 2022. Research progress on ecology of dictyostelid cellular slime molds. Mycosystema 42:160–169. doi:10.13346/j.mycosystema.220417

[7] Liu P, Zou Y, Li W, Li Y, Li X, Che S, Stephenson SL. 2019. Dictyostelid cellular slime molds from christmas Island, Indian Ocean. mSphere 4:4. doi:10.1128/mSphere.00133-19 PMC6458434 30971444

[8] Romeralo M, Baldauf S, Escalante R. 2013. Dictyostelids: evolution, genomics and cell biology. Springer, Berlin, Heidelberg.

[9] Correa-Galeote D, Roibás A, Mosquera-Corral A, Juárez-Jiménez B, González-López J, Rodelas B. 2021. Salinity is the major driver of the global eukaryotic community structure in fish-canning wastewater treatment plants. J Environ Manage 290:112623. doi:10.1016/j.jenvman.2021.112623 33901822

[10] He ZZ, Wang LT, Ge YX, Zhang SY, Tian YH, Yang X, Shu LF. 2021. Both viable and inactivated amoeba spores protect their intracellular bacteria from drinking water disinfection. J Hazard Mater 417:126006. doi:10.1016/j.jhazmat.2021.126006 33984787

[11] Raper KB. 1937. Growth and development of Dictyostelium discoideum with different bacterial associates. New Zeal J Agric Res 55:289–316.

[12] Raper KB, Smith NR. 1939. The growth of Dictyostelium discoideum on pathogenic bacteria. J Bacteriol 38:431–445. doi:10.1128/jb.38.4.431-445.1939 16560262 PMC374532

[13] Jauslin T, Lamrabet O, Crespo-Yañez X, Marchetti A, Ayadi I, Ifrid E, Guilhen C, Leippe M, Cosson P. 2021. How phagocytic cells kill different bacteria: a quantitative analysis using Dictyostelium discoideum. mBio 12:1–13. doi:10.1128/mBio.03169-20 PMC8545105 33593980

[14] Solomon JM, Isberg RR. 2000. Growth of Legionella pneumophila in Dictyostelium discoideum: a novel system for genetic analysis of host-pathogen interactions. Trends Microbiol 8:478–480. doi:10.1016/s0966-842x(00)01852-7 11044684

[15] Cosson P, Zulianello L, Join-Lambert O, Faurisson F, Gebbie L, Benghezal M, Van Delden C, Curty LK, Köhler T. 2002. Pseudomonas aeruginosa virulence analyzed in a Dictyostelium discoideum host system. J Bacteriol 184:3027–3033. doi:10.1128/JB.184.11.3027-3033.2002 12003944 PMC135065

[16] Steinert M, Heuner K. 2005. Dictyostelium as host model for pathogenesis. Cell Microbiol 7:307–314. doi:10.1111/j.1462-5822.2005.00493.x 15679834

[17] Steinert M. 2011. Pathogen-host interactions in Dictyostelium, Legionella, Mycobacterium and other pathogens. Semin Cell Dev Biol 22:70–76. doi:10.1016/j.semcdb.2010.11.003 21109012

[18] Scott TJ, Queller DC, Strassmann JE. 2022. Context dependence in the symbiosis between Dictyostelium discoideum and Paraburkholderia. Evol Lett 6:245–254. doi:10.1002/evl3.281 35784451 PMC9233174

[19] Mather RV, Larsen TJ, Brock DA, Queller DC, Strassmann JE. 2023. Paraburkholderia symbionts isolated from Dictyostelium discoideum induce bacterial carriage in other Dictyostelium species. Proc Biol Sci 290:1–10. doi:10.1098/rspb.2023.0977 PMC10354463 37464760

[20] Ayadi I, Lamrabet O, Munoz-Ruiz R, Jauslin T, Guilhen C, Cosson P. 2024. Extracellular and intracellular destruction of Pseudomonas aeruginosa by Dictyostelium discoideum phagocytes mobilize different antibacterial mechanisms. Mol Microbiol 121:69–84. doi:10.1111/mmi.15197 38017607

[21] Wang ZH, Huang W, Mai YW, Tian YH, Wu B, Wang C, Yan QY, He ZZ, Shu LF. 2023. Environmental stress promotes the persistence of facultative bacterial symbionts in amoebae. Ecol Evol 13:1–9. doi:10.1002/ece3.9899 PMC10019945 36937064

[22] Brock DA, Douglas TE, Queller DC, Strassmann JE. 2011. Primitive agriculture in a social amoeba. Nature 469:393–396. doi:10.1038/nature09668 21248849

[23] Brock DA, Callison WÉ, Strassmann JE, Queller DC. 2016. Sentinel cells, symbiotic bacteria and toxin resistance in the social amoeba Dictyostelium discoideum. Proc Biol Sci 283:283. doi:10.1098/rspb.2015.2727 PMC4855374 27097923

[24] Brock DA, Haselkorn TS, Garcia JR, Bashir U, Douglas TE, Galloway J, Brodie F, Queller DC, Strassmann JE. 2018. Diversity of free-living environmental bacteria and their interactions with a bactivorous amoeba. Front Cell Infect Microbiol 8:411. doi:10.3389/fcimb.2018.00411 30533398 PMC6266680

[25] Zou Y, Liu P, Li Y. 2021. Research progress of biological characteristics and applications of dictyostelid cellular slime molds. Mycosystema 40:294–305. doi:10.13346/j.mycosystema.190262

[26] Romeralo M, Escalante R, Baldauf SL. 2012. Evolution and diversity of dictyostelid social amoebae. Protist 163:327–343. doi:10.1016/j.protis.2011.09.004 22209334

[27] Sheikh S, Thulin M, Cavender JC, Escalante R, Kawakami S-I, Lado C, Landolt JC, Nanjundiah V, Queller DC, Strassmann JE, Spiegel FW, Stephenson SL, Vadell EM, Baldauf SL. 2018. A new classification of the dictyostelids. Protist 169:1–28. doi:10.1016/j.protis.2017.11.001 29367151

[28] Zhang ZJ, Yang YK, Zhao J, Li Y, Stephenson SL, Qiu JZ, Liu P. 2023. Environmental factors influencing the diversity and distribution of dictyostelid cellular slime molds in forest and farmland soils of western China. Microbiol Spectr 11:1–17. doi:10.1128/spectrum.01732-23 PMC10715086 37962389

[29] Zhang ZJ. 2023. Diversity of dictyostelid cellular slime molds based on different environmental factors and its relationship with symbiotic bacteria

[30] Zhang JH, Xu Y, Jin YL, Wang JY, Zhang HL, Duan WJ. 2024. Establishment and application of TaqMan probe real-time PCR method for identification of Nostoc flagelliforme. Plant Quarantine 38:38–42. doi:10.19662/j.cnki.issn1005-2755.2024.06.008

[31] Zhou CD, Huang ZS. 2018. Application of fluorescence quantitative PCR technology in the field of drug inspection. Tianjin Pharmacy 30:65–71. doi:10.3969/j.issn.1006-5687.2018.02.022

[32] Al-Zaban MI, Alrokban AH, Mahmoud MA. 2023. Development of a real-time PCR and multiplex PCR assay for the detection and identification of mycotoxigenic fungi in stored maize grains. Mycology 14:227–238. doi:10.1080/21501203.2023.2213704 37583456 PMC10424615

[33] Thieulent CJ, Carossino M, Peak L, Wolfson W, Balasuriya UBR. 2023. Multiplex one-step RT-qPCR assays for simultaneous detection of SARS-CoV-2 and other enteric viruses of dogs and cats. Viruses 15:1890. doi:10.3390/v15091890 37766296 PMC10534472

[34] Guo SQ, Fu YW, Hou TL, Huang SL, Zhang QZ. 2025. Establishment and application of TaqMan probe-based quantitative real-time PCR for rapid detection and quantification of Ichthyophthirius multifiliis in farming environments and fish tissues. Vet Parasitol 334:110381. doi:10.1016/j.vetpar.2024.110381 39742554

[35] Li KF, Wang F, Ding W, Li XL, Du CH, Yan Y, Pei XP. 2022. Application of quantitative real-time PCR in gene expression and identification of medicinal plants. World Chinese Medicine 17:3101–3111. doi:10.3969/j.issn.1673-7202.2022.21.021

[36] Li W, Sun GD, Lu JR, Zhao ZK, Yang J. 2024. Detection of adulteration of milk in special milk based on quantitative real-time PCR. Food Science:1–20. https://link.cnki.net/urlid/11.2206.TS.20241210.1326.031.

[37] Rodríguez-Zaragoza S. 1994. Ecology of free-living amoebae. Crit Rev Microbiol 20:225–241. doi:10.3109/10408419409114556 7802958

[38] Liu P, Zhang S, Zou Y, Li Z, Stephenson SL, Li Y. 2020. Distribution and ecology of dictyostelids in China. Fungal Biol Rev 34:170–177. doi:10.1016/j.fbr.2020.07.003

[39] Zhang ZJ, Yang YK, Du YJ, Zou Y, Stephenson SL, Li Y, Liu P. 2025. The community diversity and metabolic function of symbiont bacteria associated with soil amoebae (dictyostelids) in free-living habitats. Appl Soil Ecol 206:105820. doi:10.1016/j.apsoil.2024.105820

[40] Andrew A, Citartan M, Wong KA, Tang TH, Magdline Sia Henry S, Ch’ng ES. 2023. Analytical and clinical evaluation of a TaqMan real-time PCR assay for the detection of chikungunya virus. Microbiol Spectr 11:e0008823. doi:10.1128/spectrum.00088-23 37272795 PMC10433969

[41] Shahrajabian MH, Sun W. 2024. The significance and importance of dPCR, qPCR, and SYBR Green PCR kit in the detection of numerous diseases. Curr Pharm Des 30:169–179. doi:10.2174/0113816128276560231218090436 38243947

[42] Zhao X, He C, Gao D, Xu T, Li X, Liu J, Li S, Wang H. 2022. Construction of infectious cDNA clone of brassica yellows virus isolated from strawberry and establishment of TaqMan RT-qPCR. Plants (Basel) 11:3380. doi:10.3390/plants11233380 36501425 PMC9735513

[43] Lappan R, Henry R, Chown SL, Luby SP, Higginson EE, Bata L, Jirapanjawat T, Schang C, Openshaw JJ, O’Toole J, Lin A, Tela A, Turagabeci A, Wong THF, French MA, Brown RR, Leder K, Greening C, McCarthy D. 2021. Monitoring of diverse enteric pathogens across environmental and host reservoirs with TaqMan array cards and standard qPCR: a methodological comparison study. Lancet Planet Health 5:e297–e308. doi:10.1016/S2542-5196(21)00051-6 33964239 PMC8116308

[44] Wang H, Kang X, Yu L, Wang H, Müller A, Kehrenberg C, Li Y, Yue M. 2025. Developing a novel TaqMan qPCR assay for optimizing Salmonella pullorum detection in chickens. Vet Q 45:1–13. doi:10.1080/01652176.2025.2454473 PMC11784030 39882692

[45] Steele MI, Peiser JM, Shreenidhi PM, Strassmann JE, Queller DC. 2023. Predation-resistant Pseudomonas bacteria engage in symbiont-like behavior with the social amoeba Dictyostelium discoideum. ISME J 17:2352–2361. doi:10.1038/s41396-023-01535-5 37884792 PMC10689837

[46] Scott TJ, Queller DC, Strassmann JE. 2024. Complex third-party effects in the Dictyostelium-Paraburkholderia symbiosis: prey bacteria that are eaten, carried or left behind. Proc Biol Sci 291:20241111. doi:10.1098/rspb.2024.1111 39016123 PMC11253208

[47] Paquet VE, Charette SJ. 2016. Amoeba-resisting bacteria found in multilamellar bodies secreted by Dictyostelium discoideum: social amoebae can also package bacteria. FEMS Microbiol Ecol 92:1–8. doi:10.1093/femsec/fiw025 26862140

[48] Cavender JC, Raper KB. 1965. The acrasieae in nature. I. isolation. Am J Bot 52:294–296. doi:10.1002/j.1537-2197.1965.tb06788.x 14285140

[49] Gorelenkov V, Antipov A, Lejnine S, Daraselia N, Yuryev A. 2001. Set of novel tools for PCR primer design. Biotechniques 31:1326–1330. doi:10.2144/01316bc04 11768662

[50] Ye J, Coulouris G, Zaretskaya I, Cutcutache I, Rozen S, Madden TL. 2012. Primer-BLAST: a tool to design target-specific primers for polymerase chain reaction. BMC Bioinformatics 13:134. doi:10.1186/1471-2105-13-134 22708584 PMC3412702

[51] Medlin L, Elwood HJ, Stickel S, Sogin ML. 1988. The characterization of enzymatically amplified eukaryotic 16S-like rRNA-coding regions. Gene 71:491–499. doi:10.1016/0378-1119(88)90066-2 3224833

[52] Ren J, Zu C, Li Y, Li M, Gu J, Chen F, Li X. 2024. Establishment and application of a TaqMan-based multiplex real-time PCR for simultaneous detection of three porcine diarrhea viruses. Front Microbiol 15:1380849. doi:10.3389/fmicb.2024.1380849 38690365 PMC11058560

[53] Gao L, Du J, Li ZH, Xin HN, Huang JJ, Zheng DD, Li HZ, Cao XF, Cui ZF, Feng BX. 2024. A quantitative detection method for active Mycobacterium tuberculosis and its application. CN119020516A, 2024-11-26 2024

[54] Han L, Tian SG, Chen Y, Zhao JY, Chen FY, Su XT. 2022. Real-time fluorescent quantitative PCR detection kit for Candida auris and its special primers, TaqMan probe. CN110804671B, 2022-10-28 2022

[55] Liu P, Zhang ZJ, Ge JH, Zou Y, Li Z, Li S, Li Y, Stephenson SL. 2025. Dictyostelids: the second major group of slime molds. mycosphere 16:423–516. doi:10.5943/mycosphere/16/1/6

[56] Sayers EW, Cavanaugh M, Frisse L, Pruitt KD, Schneider VA, Underwood BA, Yankie L, Karsch-Mizrachi I. 2025. GenBank 2025 update. Nucleic Acids Res 53:D56–D61. doi:10.1093/nar/gkae1114 39558184 PMC11701615

[57] Thompson JD, Higgins DG, Gibson TJ. 1994. CLUSTAL W: improving the sensitivity of progressive multiple sequence alignment through sequence weighting, position-specific gap penalties and weight matrix choice. Nucleic Acids Res 22:4673–4680. doi:10.1093/nar/22.22.4673 7984417 PMC308517

[58] Kumar S, Stecher G, Tamura K. 2016. MEGA7: molecular evolutionary genetics analysis version 7.0 for bigger datasets. Mol Biol Evol 33:1870–1874. doi:10.1093/molbev/msw054 27004904 PMC8210823

[59] Minh BQ, Schmidt HA, Chernomor O, Schrempf D, Woodhams MD, von Haeseler A, Lanfear R. 2020. IQ-TREE 2: new models and efficient methods for phylogenetic inference in the Genomic Era. Mol Biol Evol 37:1530–1534. doi:10.1093/molbev/msaa015 32011700 PMC7182206

