# Supplementary material for: A novel TaqMan probe-based qPCR method for rapid detection of the bacteria-associated amoeba Heterostelium pallidum
Source: Microbiol Spectr. 2026 Apr 20;14(6):e01311-25. doi: 10.1128/spectrum.01311-25 (PMC13228067; doi:10.1128/spectrum.01311-25)
Supplement: Fig. S1 to S3; Tables S1 to S3 — Fig. S1 to S3 contain the gel images and construction of standard plasmids. Tables S1 to S3 contain the primers, probes and sequences used in manuscripts. [file spectrum.01311-25-s0001.docx]

**SUPPLEMENTAL MATERIAL**


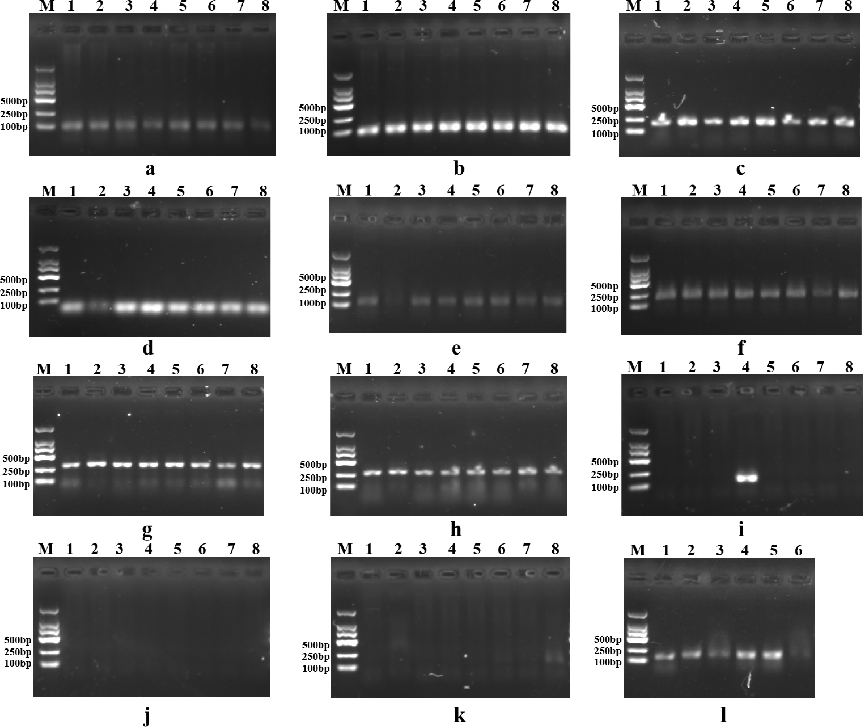


**Figure S1.** Gel image of primer gradient amplification. A: HA1F/R; B: HA2F/R; C: HA3F/R; D: HA4F/R; E: HA5F/R; F: HA6F/R; G: HA7F/R; H: HA8F/R; I: HA9F/R; J: HA10F/R; K: HA11F/R; L: HA12F/R. M: DNA marker 2000bp, A-K: 60 ℃, 59.7 ℃, 59.1 ℃, 58.1 ℃, 57 ℃, 56.2 ℃, 55.5 ℃, 55 ℃; L: 60 ℃, 59 ℃, 58 ℃, 57 ℃, 56 ℃, 55 ℃.


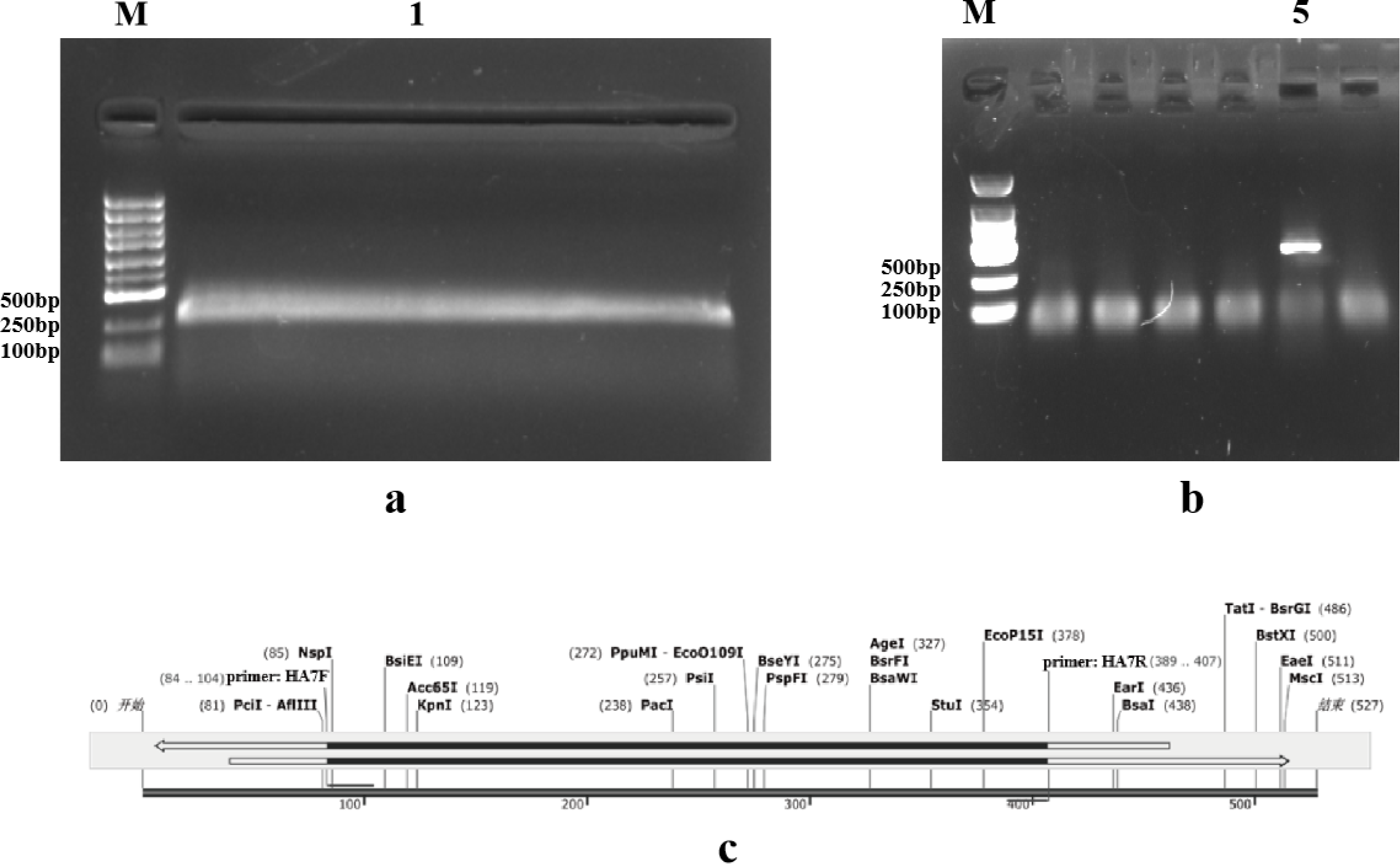


**Figure S2.** Construction of standard plasmid. a: M: DNA marker 5000bp. 1: the amplification of the genome of *Heterostelium pallidum* using specific primers HA7R/F. b: M: DNA marker 2000bp.Six transformed colonies were selected for verification. The band labeled as '5' represents the result of conventional PCR using M13 primers to validate the recombinant plasmid. The template for this PCR reaction was plasmid DNA extracted from E. coli DH5α transformants, which had previously been confirmed via colony PCR to contain the target insert. The presence of a clear single band at the expected size confirms the successful construction of the plasmid and the correct insertion of the target fragment. The band appears slightly larger than the expected insert size because it includes a portion of the vector sequence in addition to the target amplicon. c: Standard quality plasmid sequence alignment results. We performed PCR amplification and sequencing of positive transformants using the specific primers HA7F/R developed in this study. The sequencing results, aligned with the target gene fragment of *H. pallidum* (partial region of the *GAPDH* gene)


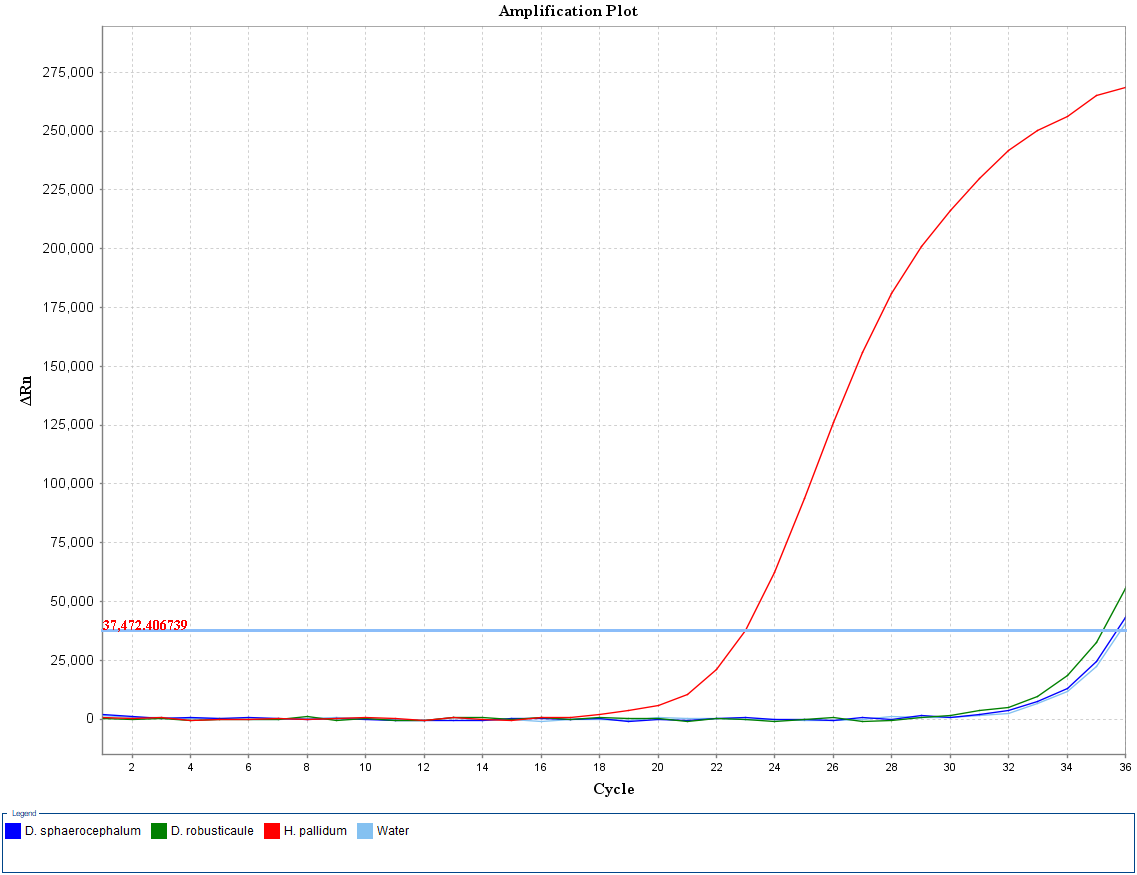


**Figure S3.** Specificity validation of the qPCR assay using DNA from pure cultures of dictyostelid species isolated from soil sample 7753. The assay yielded a strong positive signal only for *H. pallidum* (7753-2) CT = 22.94 The CT values for *D. robusticaule* (7753-1) and *D. sphaerocephalum* (7753-3) were 35.18 and 35.72, respectively, which are indistinguishable from the negative control (water, CT = 35.83) and above our detection threshold (CT > 35).

| Primer Pair | Amplicon Size (bp) | Primer Name | Sequence(5’ -3’) | Target Gene |
| --- | --- | --- | --- | --- |
| HA1 | 83 | HA1F | ATCAGGTGCTGGTAACAAT | *β-tubulin* |
|  |  | HA1R | CGACGAATGACATCCAATAC | *β-tubulin* |
| HA2 | 82 | HA2F | AAGGTATGGAAGAGTTAGAGTT | *β-tubulin* |
|  |  | HA2R | GATGTCGTTGTATTGTTGGTA | *β-tubulin* |
| HA3 | 176 | HA3F | GACCTACAAGCCAGAAGT | *GAPDH* |
|  |  | HA3R | CTCCAGTCCTTACCAGATG | *GAPDH* |
| HA4 | 24 | HA4F | TTTCGCTGAGTATGACCC | *GAPDH* |
|  |  | HA4R | GTTAGCTGGGTCATACTCA | *GAPDH* |
| HA5 | 111 | HA5F | TCTCCGTGCTTCAATCGAGA | *GAPDH* |
|  |  | HA5R | ACGACCGTGAACTGAATCGT | *GAPDH* |
| HA6 | 117 | HA6F | TCGTTTCAAGGGTACCGTCAG | *GAPDH* |
|  |  | HA6R | AGCACCAACTGATCCCCATT | *GAPDH* |
| HA7 | 324 | HA7F | TGTTCTCATACGATTCAGTTC | *GAPDH* |
|  |  | HA7R | AGATGGAGCAGAGATGATG | *GAPDH* |
| HA8 | 196 | HA8F | TTCAAGGGTACCGTCAGCAA | *GAPDH* |
|  |  | HA8R | ATGACCTTCTTGGCACCACC | *GAPDH* |
| HA9 | 133 | HA9F | ATCAACATTACAAGAAGCAAGA | *RAD50* |
|  |  | HA9R | TCAAGTGGAGAGCATCAAT | *RAD50* |
| HA10 | 137 | HA10F | GAAAACTCATAAAACAATAGAAA | *RAD50* |
|  |  | HA10R | GGAATGGCATAAAGTATATC | *RAD50* |
| HA11 | 128 | HA11F | ATCACCATCTGTAGTAGT | *RAD50* |
|  |  | HA11R | TTACCAATTTCTATTGTTTTATG | *RAD50* |
| HA12 | 133 | HA12F | ATCAACATTACAAGAAGCAAGA | *RAD50* |
|  |  | HA12R | TCAAGTGGAGAGCATCAAT | *RAD50* |
| Probe | N/A | HA7-TaqMan | FAM-ACCGTCAGCAAGGATAACGAAGGT-TAMRA | *GAPDH* |

**Table S1**. Primers and probes used in this study

**Table S2.** Sequences used in phylogenetic analysis based on SSU.

| **Species** | **Strain Number** | **SSU Accession Numbers** |
| --- | --- | --- |
| *Acytostelium amazonicum* | HN1B1 | HQ141511.1 |
| *A. anastomosans* | PP1 | AM168115.1 |
| *A. digitatum* | OH517 | AM168114.1 |
| *Cavenderia amphispora* | BM9A | HQ141521.1 |
| *C. antarctica* | NZ43B | AM168080.1 |
| *C. aureostabilis* | TH10B | MH745571.1 |
| *C. delicata* | TNS-C-226 | AM168093.1 |
| *Coremiostelium polycephalum* | Landolt #1675 GUAM | HQ141490.1 |
| *Dictyostelium ammophilum* | KBK4A | HQ141478.1 |
| *D. discoideum* | NC4 | AM168071.1 |
| *D. macrocephalum* | B33 | AM168049.1 |
| *D. medium* | TNS-C-205 | AM168050.1 |
| ***D. robusticaule*** | **7753-1** | **PV478263** |
| *D. robusticaule* | 5729-bai-2021 | MW931857.1 |
| *D. robusticaule* | 5729-huang-2021 | MW931856.1 |
| ***D. sphaerocephalum*** | **7753-3** | **PV478265** |
| *D. sphaerocephalum* | Boots 01 A1 | JN590752.1 |
| *D. sphaerocephalum* | Boots 07 A2 | JN590754.1 |
| *Hagiwaraea rhizopodia* | AusKY-4 | AM168063.1 |
| *Heterostelium equisetoides* | B7JB | AM168099.1 |
| *H. tenuissimum* | TNS-C-97 | AM168105.1 |
| *H. tikalense* | OH595 | AM168106.1 |
| ***H. pallidum*** | **7753-2** | **PV478264** |
| *H. pallidum* | TNS-C-98 | AM168103.1 |
| *H. pallidum* | WS-1 | LC159252.1 |
| *H. pallidum* | WS-20 | LC159270.1 |
| *Polysphondylium violaceum* | 209 | HQ141486.1 |
| *Rostrostelium ellipticum* | AE2 | AM168112.1 |
| *Raperostelium maeandriforme* | OH604 | JF892719.1 |
| *R. ohioense* | Okla4C | HQ141493.1 |

**Table S3.** **Assessment of PCR inhibition in soil DNA extracts.**

| **Simpore** | **CT±SD** |
| --- | --- |
| plasmid standard&water | 30.15 ± 0.18 |
| plasmid standard&7082 | 30.31 ± 0.22 |
| plasmid standard&7858 | 30.26 ± 0.15 |
| water | 35.88 |
